# Promoting menstrual health among persian adolescent girls from low socioeconomic backgrounds: a quasi-experimental study

**DOI:** 10.1186/1471-2458-12-193

**Published:** 2012-03-15

**Authors:** Moloud Fakhri, Zeinab Hamzehgardeshi, Nayereh Azam Hajikhani Golchin, Abdulhay Komili

**Affiliations:** 1 Member of faculty, Department of Midwifery, Mazandaran University of Medical Sciences, Sari, Iran; 2 PhD candidate, Department of Reproductive Health, Tehran University of Medical Sciences, Tehran, Iran; 3 Member of faculty, Department of Midwifery, Islamic Azad University Gorgan branch, Gorgan, Iran; 4 Academician of Academy of pedagogical and social sciences of Russian Federation, Moscow, Russia

## Abstract

**Background:**

Research in the past decade has revealed average to poor menstrual health among many Iranian girls. The present study investigated the effectiveness of a health promotion project on improving menstrual health in adolescent girls in Iran.

**Methods:**

A quasi-experimental study was conducted to evaluate the effectiveness of the health intervention program. A total of 698 students (study participants and controls) in several schools in Mazandaran province, Iran were included. The project comprised 10 two-hour educational sessions. Educational topics included the significance of adolescence, physical and emotional changes during adolescence, pubertal and menstruation health and premenstrual syndrome. A self-administered questionnaire measuring demographic characteristics, behaviors during menstruation, menstrual patterns, sources of information about menstruation and personal health data was administered. The questionnaire was administered to all participating students after the experimental group received the training.

**Results:**

Among the most significant results was the impact of educational sessions on bathing and genital hygiene. A total of 61.6% in the experimental group compared with 49.3% in the control group engaged in usual bathing during menstruation (p = 0.002). Individual health status was significantly statistically correlated with menstrual health. Attitude towards menstruation was also significantly related to menstrual health.

**Conclusions:**

The present study confirms that educational interventions, such as the health promotion project in this study, can be quite effective in promoting menstrual health.

## Background

Adolescence is considered a critical period in human evolution, although it is often not recognized as such by health care workers and parents as well as professionals in adult medicine and pediatric disciplines [1]. The onset of menstruation in adolescence is a phenomenon that signals reproductive maturity and should not be seen as an abnormal condition or disease. Adolescent girls often do not receive accurate information about menstrual health because of culturally specific practices that lead to incorrect and unhealthy behaviors [2].

In recent years, reproductive health care has been a primary concern of the Ministry of Health and Medical Education and the Ministry of Education in Iran. In this regard, the Department of Youth and School Health started a reproductive health care program in January 2005 to accompany the United Nations Population Fund programs. Education regarding menstrual health has changed since 2004 in Mazandaran province. In 2006, in addition to routine education under the supervision of the Deputy of Provincial Health taking place in schools, education about hygiene during menstruation was introduced to target groups including students, parents and school personnel. Subsequently, the education administration of Mazandaran province began conducting health promotion projects focused on puberty. The puberty health education is running in some schools now.

Research worldwide has addressed issues related to menstrual health. Studies have revealed the effectiveness of health education interventions conducted among high school, guidance school and even primary school girls [3-6]. A study in Nepal [7] reported that knowledge and practices related to menstruation were not satisfactory. These researchers suggested that health education by teachers, parents and the media is important in addressing misconceptions about menstrual health.

Changes in the health education system to promote reproductive health have become a priority for the Ministry of Health and Medical Education and the Ministry of Education. This is likely to result in a change in hygiene patterns during menstruation for girls participating in the program.

Research related to menstrual hygiene programs in the past decade suggests average to poor menstrual health among Iranian girls [1,2,8]. One study reported that although female adolescents in Mazandaran province had some knowledge of puberty, most of them did not fully understand it and lacked accurate scientific knowledge about their physiological maturity [9]. The present study investigated the effect of education to improve menstrual health through a health promotion project for several schools in Mazandaran province.

## Methods

A quasi-experimental study was conducted to evaluate the personal health and hygiene of high school girls during menstruation.

An experimental study is a research design which researcher manipulates one or more variables, and measures any change in other variables. It includes pre-post test, a study group and a control group, and random sampling methods. A quasi-experimental study is lack one or more of these design characters.

Ethical approval was obtained from the Ethics Committee of the Mazandaran University of Medical Sciences (code number: 87-10-16-87-92) for this research project. Informed consent was obtained from parents of all teenagers who participated in the study.

The health promotion initiative by the education organization in Mazandaran province, in north of Iran, was conducted in selected high schools. The project included 10 two-hour educational sessions using adolescent health resources. The educational manual was developed by adolescent health professionals' team. Educational topics included the significance of adolescence, physical and emotional changes during adolescence, pubertal and menstruation health and premenstrual syndrome. Participants comprised 689 high school girls (349 in the study group and 349 in the control group) 14 to 18 years of age in urban and rural public high schools with low socio economic status in Mazandaran province. The control group comprised high school students in Mazandaran province who did not participate in the education. As much as possible, the control samples were selected from the nearest neighbor school.

Factors matched in the study and control group were school type (rural or urban), grade, age and educational field of study. Inclusion criteria were students in high school, 14 to 18 years of age, parental consent for participating in the project and having had at least one menstrual period. Sampling was done in cooperation with the Ministry of Education in Mazandaran province and related administrations.

A questionnaire developed by the researcher was used for data collection. The menstrual health education was a government-sponsored program. The Youth and School Health Department delivered the training and evaluated menstrual health status before the education. Menstrual health was considered poor or average in Mazandaran province. The questionnaire in the present study was administered by the researcher to both the experimental and control group after the education. This self-administered questionnaire comprised 71 items in five sections: demographic characteristics, behaviors during menstruation, menstrual-related patterns, sources of information about menstruation and questions related to personal health.

We determined the validity and reliability of the questionnaire as follows. Face and content validity were determined based on viewpoints of the adolescent girls, health sciences specialists, and experts in the community. Test-retest reliability was examined using a 10-14 day interval with 20 qualified and available female students. These students were not part of the study. The questionnaire was reviewed and analyzed for repeatability and internal consistency aspects. Cronbach's alpha coefficient was used to assess internal consistency. Repeatability was estimated using the intra-class correlation coefficient (ICC). Cronbach's alpha coefficient and mean ICC were 0.88, and 0.90, respectively.

Based on ICC reliability ranges of less than 0.4 (poor), 0.4-0.7 (fair to good), 0.6-0.8 (good) and 0.8-1 (excellent), the reliability of the questionnaire was considered excellent [10].

Menstrual health was measured by investigating variables such as the pad material used, daily use of a clean pad, number of pad replacements during 24 hours, changing the pad at night or when in school, tendency to stay at home or not exercise, ability to properly perform daily tasks, negative impact of menstruation on studying, frequency of school absences during menstruation, noting the first day of the monthly menstruation, noticing typical period occurrence, paying attention to sudden changes in menstruation, changes in diet during menstruation including eating more protein and less salt and sugar, reduced consumption of snacks, sweets or pickles and increased consumption of vegetables, fruits and grains, avoiding certain foods, iron supplements during menstruation and hygiene habits including bathing the genitals.

The socio-economic level was categorized into four groups, based on the occupation of the father of the family: the higher class, including major landowners, merchants and manufacturers; the middle class, including government administrators, teachers, minor landowners, army officers, clergymen, individuals with personal professions and professionals; the working class, including professional and skilled workers; and the lower class, including the unemployed and the unskilled workers.

It is noteworthy that scores 0 and 1 were allocated to each of the questions of menstrual health and individual health status. Adolescent were arrived at percentile 0-25 (poor), 25-50 (average), 50-75 (good), and 75-100 (excellent).

The data collected were analyzed using the Statistical Package for the Social Sciences version 16 for Windows (SPSS Inc., Chicago, IL, USA). Descriptive and analytical indicators were determined. T-tests and chi square tests were employed for analysis, and the level of significance was set at 0.05.

## Results

The average age of the girls in this study was 15.7 years (SD = 1.08). A total of 35.9% were from urban areas, and 95.8% were from Mazandaran province originally. The socioeconomic level was lower class for 86.2% of the participants. For 53.2%, their family had more than 5 members. Regarding parental education, 77.5% of fathers did not have a diploma, and 11.6% held an academic degree. Of the mothers, 87.6% did not have a diploma, and 4.1% had an academic degree.

As shown in Table 1, the level of menstrual health of subjects who participated in the puberty health promotion program was significantly better than that of the control group (p = 0.013).

**Table 1:** Comparison of menstrual health between the experimental group and control group

Menstrual Health	Experimental Group n (%)	Control Group n (%)
Good and Excellent	30(8.6)	17(4.9)

Average	299 (85.7)	295 (84.5)

Poor	20 (5.7)	37 (10.6)

Total	349 (100)	349 (100)

Among the most significant results was the impact of participation in the classes on bathing and genital hygiene. A total of 61.6% in the experimental group compared with 49.3% in the control group engaged in usual bathing during menstruation (p = 0.002). There was a statistically significant difference between those who bathed in the first two days of menstruation in the experimental and control groups, 19.5% and 12.5%, respectively (p = 0.01). Only 19.7% of study participants compared with 25.3% of controls always or sometimes avoided washing their genitals after urinating during menstruation (p = 0.02). Regarding educational field of study, 12.3% of students in the experimental sciences had good genital hygiene during the menses compared to 6% for math and physics students and 5.5% for students in the humanities (p = 0.007).

Regarding the relationship between menstruation patterns and level of menstrual health, premenstrual syndrome (PMS) and menstrual cycle regularity were statistically among the most significant factors. PMS was reported by 9% of the participants. Among the students with irregular menstrual cycles, 10.4% had poor menstrual health compared with 5.5% among students with regular cycles (p = 0.001). The students with PMS had poorer menstrual health (17.5%) than those not suffering from PMS (7.2%, p = 0.01).

Individual health status was also statistically related to menstrual health. Those with better individual health behaviors had better menstrual health (p = 0.001) (Table 2).

**Table 2:** Relationship between menstrual health and individual health status

Individual Health Status	Menstrual Health		
	
	Good and Excellent (%)	Average (%)	Poor (%)
Good and Excellent	18 (38.3)	60 (10.1)	2 (3.5)

Average	24 (51.1)	355 (59.8)	32 (65.1)

Poor	5 (10.6)	179 (30.1)	23 (40.4)

Total	47 (100)	594 (100)	57 (100)

Attitude towards menstruation was related to menstrual health. A total of 78.7% of participants with good and excellent menstrual health considered menstruation a natural phenomenon and an indication of mental and physical health. For 6.4% of participants, it was troublesome, disturbing and agonizing, and 8.5% expressed both views. The remaining participants expressed neither opinion. These four views (i.e., positive, troublesome, both and no opinion) among the participants with average menstrual health represented 50.8%, 13%, 19.4% and 16.9%, respectively, and among those with poor menstrual health 33.3%, 15.8%, 35.1% and 15.8%, respectively (p = 0.0001). A total of 38.3% with high, 22.2% with average and 24.6% with poor menstrual health felt positive about menstruation; 40.4% with high, 39.7% with average and 28.1% with poor menstrual health had no opinion; and 10.6% with high, 32.2% with average and 40.4% with poor menstrual health felt it was troublesome (p = 0.01.). Sources of information about menstruation were mainly mothers (24.5%), health care professionals at clinics (9.3%), friends (8.7%), elder sisters (7.7%), other family members (3%), books and magazines (2.7%), mass media (1%) and other.

## Discussion

The results showed a statistically significant difference between menstrual health of the experimental group compared with the control group, providing support for participation in adolescent health care programs [6,11,12]. Poor menstrual hygiene is a risk factor for reproductive tract infections [11]. Thus, health care during menstruation is a vital element of any health program for girls during puberty. Health programs on television, programs by health care professionals in schools and trained parents can play a major role in transmission of knowledge related to good menstrual health.

Chang et al. [6] conducted a similar study of primary school girls in grades 5 and 6 and showed that educational programs in schools for students and their parents are quite effective in increasing menstrual health.

A study in Egypt [4] showed the efficacy of a menstrual education program for first and second year girls at a secondary school and recommended expanding the program to elementary, preparatory and other secondary schools.

The above studies confirm that health education programs in schools can intervene effectively to improve menstrual health. Research studies in Iran, in contrast to international studies that consider menstrual health satisfactory or somewhat poor, reported menstrual health as average or poor prior to 2004. Researchers in Iran investigated 250 high school girls in the city of Karaj in 1998 and found that only 6% practiced menstrual hygiene such as bathing and using hygienic materials. Forty-eight percent avoided any physical activity or exercise during menstruation. A total of 67% reported having taken medicine for pain without consulting a doctor. Furthermore, their background knowledge had no impact on their menstrual health because it was insufficient and not completely accurate [2].

According to our results and similar studies, adolescents get most of their information about menstruation from their mothers [1,12]. Salari et al. [1] investigated knowledge, attitudes and practices related to menstrual health of high school girls in the city of Urmia, Iran in 1999-2000. The results showed that 68.8% got menstruation information from their mothers and sisters, and 64.1% reported a need for further knowledge. Statistically, there was a significant relationship between knowledge and menstrual health of the subjects and the mothers' occupation.

The results of a similar study done in the north and south of Tehran showed that menstrual health is not related to socioeconomic level of the family. Having an elder sister in the family had no significant relationship with the level of menstrual health. We propose that the absence of health education in schools is an important factor affecting menstrual health in adolescent girls [13]. Various studies have shown that health education increases knowledge and positive attitudes towards puberty, and facilitates acceptance of pubescent changes as a natural physiological phenomenon [12].

## Conclusion

The present study confirms that the health promotion project in Mazandaran province has been quite effective in promoting menstrual health among high school girls, especially in deprived regions that are the target groups in any health promotion project. Similar studies and educational programs sponsored by the Ministry of Education and health clinics would be of value. In addition, future studies to support the results of the present study regarding the benefits of direct education intervention are recommended.

## Competing interests

The authors declare that they have no competing interests.

## Authors' contributions

All four authors contributed to the development of ideas and design of the study. MF wrote the first draft of the manuscript, which was critiqued by the other authors. The final version of the manuscript was critically reviewed by ZH. All authors read and approved the final manuscript.

## Pre-publication history

The pre-publication history for this paper can be accessed here:

http://www.biomedcentral.com/1471-2458/12/193/prepub
